# Evaluation of the Efficacy of Oral and Intramuscular Administration of Dexamethasone on Postoperative Pain, Swelling, and Trismus After Surgical Removal of Impacted Third Molar: A Comparative Split-Mouth Study

**DOI:** 10.7759/cureus.38306

**Published:** 2023-04-29

**Authors:** Kinjal S Lakhani, Samir Joshi, Sudhir Pawar, Vivek S Nair, Vaishali Korrane, Hamza Salema, Nayla Khan, Jay Patel

**Affiliations:** 1 Oral and Maxillofacial Surgery, Bharati Vidyapeeth Dental College and Hospital, Pune, IND; 2 Oral Medicine and Radiology, Bharati Vidyapeeth Dental College and Hospital, Pune, IND

**Keywords:** dexamethasone, quality of life, impacted mandibular third molars, pain control, minor surgical procedure, oral corticosteroids

## Abstract

Context

Over the past 60 years, several researchers have conducted extensive studies on the use of dexamethasone to reduce the postoperative complications of lower third molar surgery, namely, pain, edema, and trismus. In this study, we compared the oral and intramuscular methods of dexamethasone administration.

Purpose

The aim of this research was to assess pain, edema, and trismus in the postoperative period following the surgical removal of the lower third molar using 8 mg of dexamethasone given orally or by intramuscular injection.

Method

A split-mouth technique was employed for the study, in which each of the two bilaterally impacted mandibular third molars was removed one at a time, separated by at least two weeks. There were 26 participants in this experiment. Two groups were created from the research sample: group A (injection dexamethasone) and group B (tablet dexamethasone). The pain was assessed on the first, second, and third postoperative days. On the first, third, and seventh postoperative days, the parameters, such as edema and trismus, were evaluated.

Results

As per our study, in terms of edema and trismus, there was less of a statistically significant difference between the two interventions at all time points. While the pain score had a significant difference between both interventions.

Conclusion

Hence, we conclude that oral dexamethasone is an effective alternative to intramuscular dexamethasone. Oral dexamethasone is comparatively simple, less invasive, painless, and easy for the surgeon and for apprehensive patients, and it offers a cost-effective solution for the suffering often associated with the extraction of impacted lower third molars.

## Introduction

The transalveolar extraction of the impacted third molars is one of the most frequently encountered minor surgical procedures in oral surgery. This procedure is often associated with considerable postoperative sequelae such as pain, edema, and trismus, which often cause discomfort to the patient [1]. According to the literature, postoperative edema may be due to the release of prostaglandins and other inflammatory mediators from the phospholipid membrane as a result of surgical trauma [2].

The use of corticosteroids is an effective method that can alleviate the aforementioned postoperative sequelae. Classification of corticosteroids is based on their duration of action. Short-acting glucocorticoids have a duration of action of less than 12 hours, they include hydrocortisone and cortisone. Intermediate-acting glucocorticoids include prednisone and prednisolone with a duration of action of about 12-36 hours. Long-acting glucocorticoids include dexamethasone and betamethasone, with a duration of action greater than 36 hours [3].

Dexamethasone is one of the most potent synthetic corticosteroids with a much greater anti-inflammatory effect and minimal unfavorable impact on leukocyte chemotaxis [4]. It has a plasma half-life of 100-300 minutes with a biological half-life of 36-72 hours [5]. Oral surgeons have been using dexamethasone since 1965 [6,7]. Evidence suggests that the clinical use of dexamethasone should be in moderation and for a limited time because, according to the endocrinology analysis, after the fifth day of use, the therapy starts producing immunosuppression [8,9].

There appears to be solidarity regarding the benefits of prophylactic antibiotics and the use of corticosteroids in decreasing postoperative complications after the third molar surgery [10,11].

This study compares the effect of intramuscular (IM) administration of dexamethasone (8 mg) in the deltoid muscle with that of oral dexamethasone (8 mg) on postoperative pain, swelling, and trismus after surgical removal of bilateral impacted mandibular third molars having similar difficulty indices.

## Materials and methods

This study was conducted on 26 healthy patients (14 males and 12 females) with an average age of 18 years (range = 15-30 years), with bilateral lower third molars in similar positions and with similar difficulty index, for which surgical removal had been indicated. Institutional Review Board and Ethics Committee’s approvals were obtained for conducting this study. The subjects were selected keeping in mind the following inclusion criteria: no history of allergy to dexamethasone, amoxicillin, or acetaminophen and a surgical site free of active infection. This study was conducted by a double-blind split-mouth technique wherein removal of the impacted mandibular third molars was done one at a time separated by a gap of at least two weeks.

All patients were given preoperative antibiotics two hours prior to the surgery. Patients were given an information sheet and were explained in detail regarding the procedure and study. Written informed consent was taken from the patient before the procedure for publication of the study findings. A detailed case history was obtained from all the patients participating in the study, and standard sterilization protocol was followed throughout the study. All the patients were explained about the visual analog scale preoperatively, which was filled by the patients based on their experience postoperatively. All injections and third molar surgery were performed by the same surgeon and all the parameters were recorded by the same clinician. The same conventional surgical method was used for all lower third molar procedures, under the usual inferior alveolar nerve block and buccal nerve block. Painting and draping of the patient were done under an aseptic protocol. Local anesthesia with adrenaline in 1:200,000 was administered and inferior alveolar, lingual, and long buccal nerve blocks were given. An appropriate incision was taken, and the flap was reflected. The impacted tooth was sectioned and surgically removed. Flap was sutured with resorbable (3-0) Vicryl sutures (manufactured in India by Johnson & Johnson Ltd, Mumbai). Each patient had to undergo two surgical extractions with an interval of at least two weeks.

In the first appointment, the patient was given 8 mg 2 ml dexamethasone injection intramuscularly into the deltoid muscle following surgical removal of the impacted mandibular third molar. While in the second appointment, the surgical removal of the mandibular third molar was carried out on the same patient on the contralateral side and 8 mg of dexamethasone was given per oral immediately following the surgery. Given that the usual daily output of cortisol is 15 to 25 mg, but that it may be produced up to 300 mg during a crisis, and that the 8 mg of dexamethasone is nearly equivalent to this quantity of released cortisol, 8 mg of oral dexamethasone was the lowest dose with the best results that could be attained [12]. Dexamethasone's onset of action is thought to be between one and two hours, giving it adequate time to diffuse through the cell membrane. The first 24 hours following surgery are said to be when corticosteroids are working at their peak capacity, with the effects perhaps continuing for three days. Inflammation, a defensive response, is the primary cause of postoperative swelling and edema. Pain and swelling that are indicative of inflammation develop gradually. Dexamethasone is thus given immediately in the postoperative period to decrease these inflammatory reactions [4]. The wash-out period between the two procedures was two weeks.

**Figure 1:**
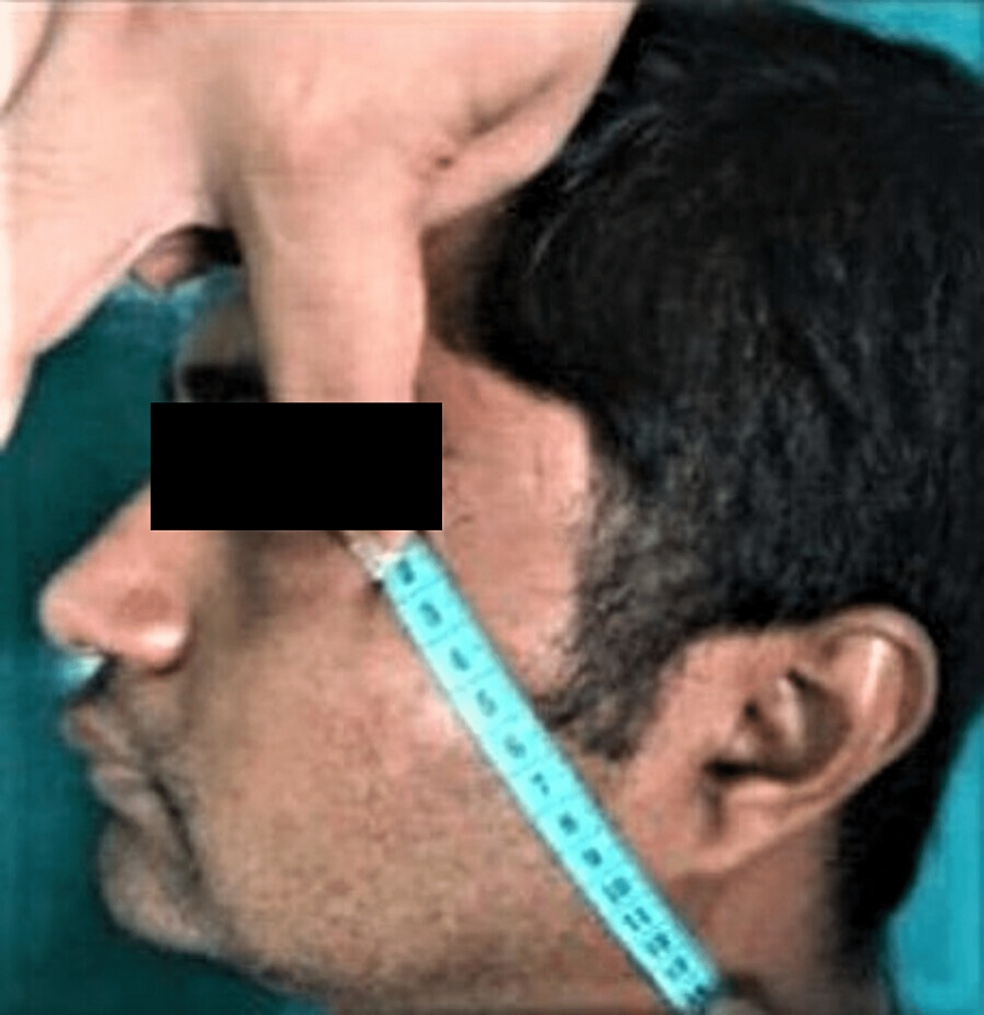
Line 1: measurement from lateral canthus of the eye to soft tissue gonion

Parameters such as swelling and trismus were recorded for the patients after surgical removal of bilaterally impacted mandibular third molars on the first, third, and seventh days postoperatively. The pain was evaluated on the first, second, and third days postoperatively (morning and evening). The swelling was measured using Matsumara and Gabka technique (in mm) with the help of a standard measuring tape from the lateral canthus of the eye to soft tissue gonion (LCG) (line 1), as shown in Figure 1.

**Figure 2:**
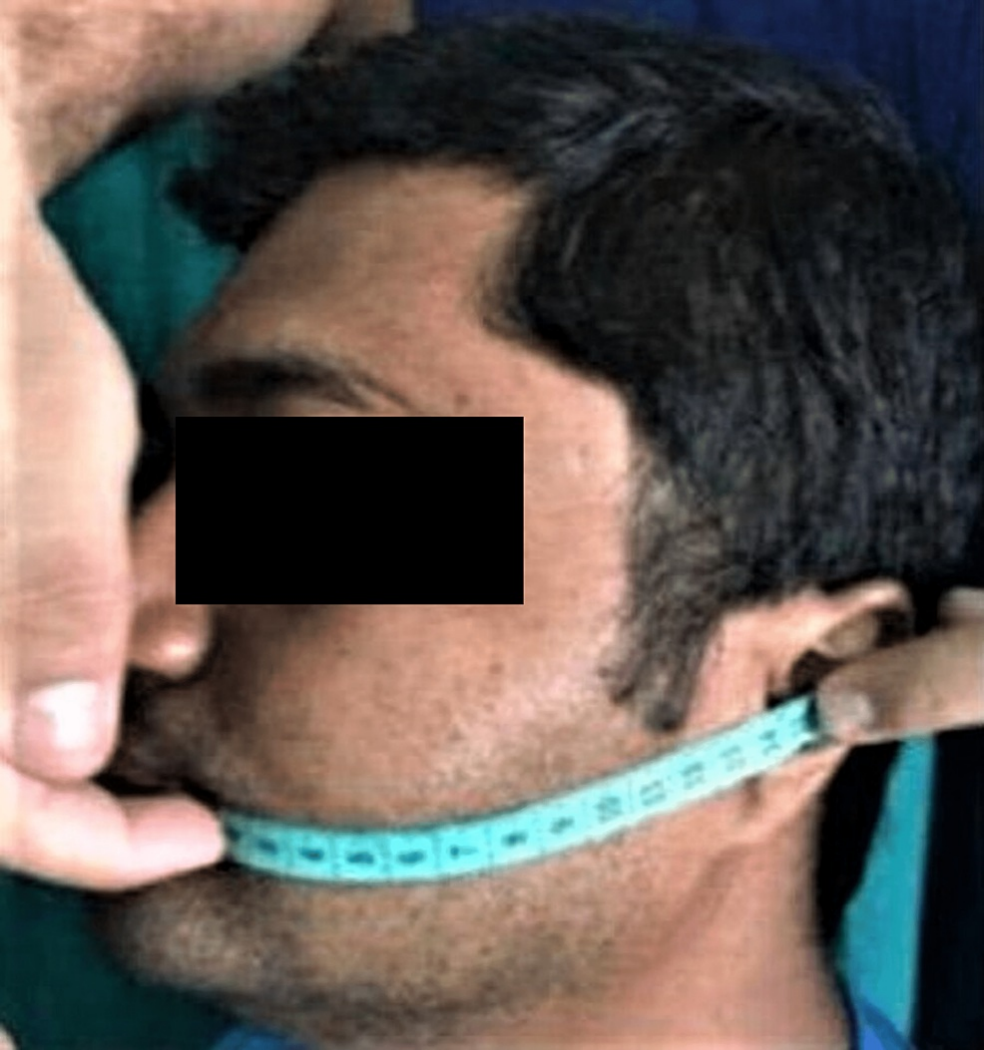
Line 2: measurement from the corner of the mouth to the tragus of the ear

The second measurement for swelling was from the tragus to the corner of the mouth (TM) (line 2), as shown in Figure 2.

**Figure 3:**
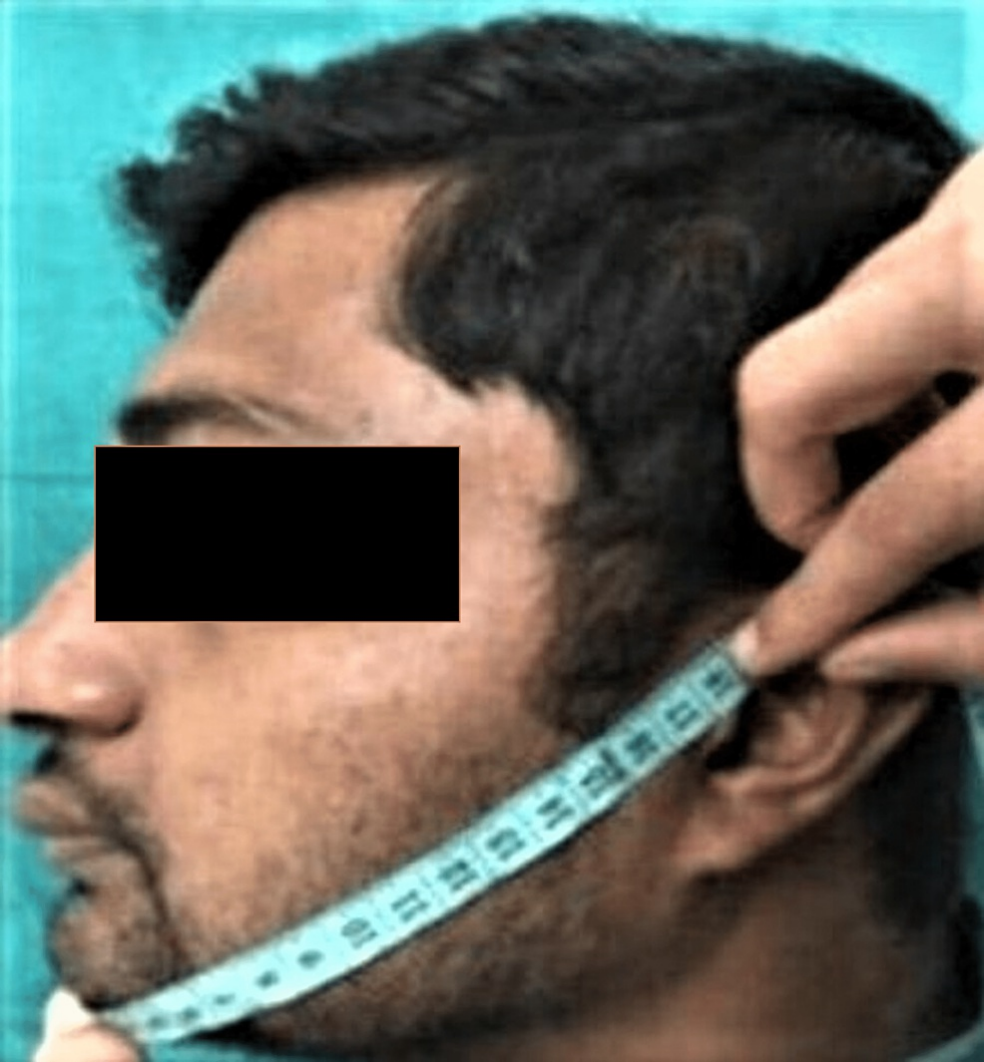
Line 3: measurement from the tragus of the ear to soft tissue pogonion

And the third imaginary line was from the tragus to the soft tissue pogonion (TP) (line 3), as shown in Figure 3.

The preoperative measurements were used as baseline values. The difference between each postoperative evaluation and baseline indicated the swelling for that day. To eliminate the observer bias, only one observer measured the swelling in all the patients.

Postoperative average pain was assessed over the next first, second, and third postoperative days once in the morning and at bedtime using a 10-point visual analog scale (VAS) with a graphic rating scale marked by the patient herself or himself as per the pain experienced by the patient. This is a numeric rating scale on which patients rate their current pain intensity, ranging from “0” (no pain) to “10” (worst possible pain).

**Figure 4:**
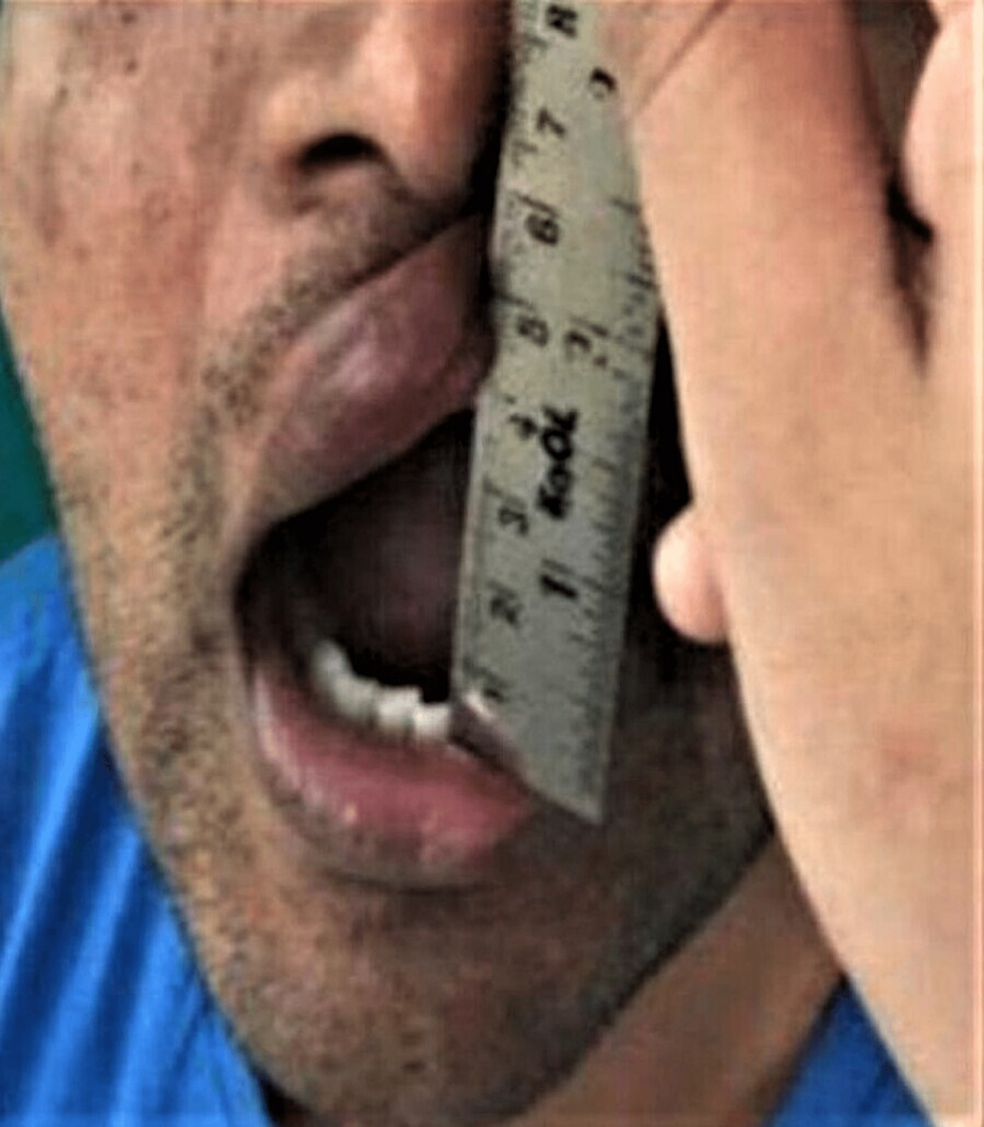
Measurement of the interincisal distance

Trismus was evaluated by measuring the inter-incisal distance at maximum mouth opening (mm) using a calibrated scale between maxillary and mandibular incisal edges, as shown in Figure 4. It was recorded on the day of the surgery (preoperative), and postoperatively on the first, third, and seventh postoperative days in both groups. The difference in interincisal opening pre and postoperatively indicated trismus.

All the patients were given the same postoperative instructions and a course of antibiotics, i.e., tablet Augmentin 625 mg, which contains 500 mg amoxicillin + 125 mg clavulanic acid, and analgesic tablet Enzoflam, which contains diclofenac 50 mg + paracetamol 325 mg + serratiopeptidase 15 mg, following the surgical removal of impacted mandibular third molars for three days. The patient’s data were statistically analyzed. The level of significance used in the statistical decisions was p < 0.05.

## Results

The mean value of facial swelling at line 1 for intramuscular dexamethasone on day one was 11.096 cm, and for oral dexamethasone, it was 12.392 cm, and the results were statistically significant. For day three, the mean for intramuscular dexamethasone was 9.826 cm, and for oral dexamethasone, it was 11.623 cm, and their difference using the independent t-test was found to be statistically significant as well. The result remained consistent for day seven, with the mean for intramuscular dexamethasone being 9.203 cm and for oral dexamethasone being 11.084 cm, and it was statistically significant too. The result implies that the facial swelling at line 1 was almost identical at all time points for the intramuscular dexamethasone and the oral dexamethasone intervention group (Table 1).

**Table 1:** Comparison of facial swelling (in cm) at line 1 between intramuscular dexamethasone and oral dexamethasone on the first, third, and seventh day postoperatively using the independent t-test

Postoperative evaluation times	Intramuscular dexamethasone		Oral dexamethasone		p-value
	Mean	Std. Deviation	Mean	Std. Deviation	
1^st^ day	11.096	1.098	12.392	1.057	0.000
3^rd^ day	9.826	1.001	11.623	0.993	0.000
7^th^ day	9.203	0.947	11.084	0.985	0.000

The mean value of facial swelling at line 2 for intramuscular dexamethasone on day one was found to be 13.265 cm, and for oral dexamethasone, it was 14.392 cm, and the results were statistically significant using the independent t-test. For day three, the mean for intramuscular dexamethasone was 11.896 cm, and for oral dexamethasone, it was 13.557 cm, and their difference was statistically significant. Similarly, for day seven, the mean for intramuscular dexamethasone was 11.176 cm, and for oral dexamethasone, it was 13.107 cm, and the result was statistically significant as well. It is pretty evident from the result that the facial swelling at line 2 for both interventions was almost identical, placing them at a similar effectiveness level (Table 2).

**Table 2:** Comparison of facial swelling (in mm) for line 2 between intramuscular dexamethasone and oral dexamethasone on the first, third, and seventh day postoperatively using the independent t-test

Postoperative evaluation times	Intramuscular dexamethasone		Oral dexamethasone		p-value
	Mean	Std. Deviation	Mean	Std. Deviation	
1^st^ day	13.265	1.126	14.392	0.905	0.000
3^rd^ day	11.896	0.792	13.557	0.845	0.000
7^th^ day	11.176	0.867	13.107	1.001	0.000

An independent t-test was used to compare the mean values of facial swelling at line 3 between intramuscular dexamethasone and oral dexamethasone. For day one, the mean value for intramuscular dexamethasone was found to be 15.019 cm, and for oral dexamethasone, it was 16.215 cm. For day three, the mean for intramuscular dexamethasone was 13.776 cm, and for oral dexamethasone, it was 15.442 cm. Finally, for day seven, the mean for intramuscular dexamethasone was 13.061 cm, and for oral dexamethasone, it was 14.869 cm. All the aforementioned results were found to be statistically significant. In terms of swelling at line 3, there was not much difference between intramuscular dexamethasone and oral dexamethasone, which is indicative of both of them being equally effective (Table 3).

**Table 3:** Comparison of facial swelling (in cm) for line 3 between intramuscular dexamethasone and oral dexamethasone on the first, third, and seventh day postoperatively using the independent t-test

Postoperative evaluation times	Intramuscular dexamethasone		Oral dexamethasone		p-value
	Mean	Std. Deviation	Mean	Std. Deviation	
1^st^ day	15.019	1.218	16.215	0.673	0.000
3^rd^ day	13.776	0.881	15.442	0.809	0.000
7^th^ day	13.061	0.969	14.869	1.059	0.000

The mean value of mouth opening for intramuscular dexamethasone on day one was 32.076 mm, and for oral dexamethasone, it was 30.423 mm, and the difference between them using the independent t-test was statistically significant. Similarly, for day three, the mean for intramuscular dexamethasone was 33.692 mm, and for oral dexamethasone, it was 32.692 mm, and their difference was found to be statistically significant as well. However, for day seven, although the mean for intramuscular dexamethasone was 38.538 mm, and for oral dexamethasone, it was 37.653 mm, the result was not found to be statistically significant. The mean values for both intervention groups are very close to each other, which can imply that both of them can be used with similar expected efficacy (Table 4).

**Table 4:** Comparison of mouth opening (in mm) indicative of trismus between intramuscular dexamethasone and oral dexamethasone on the first, third, and seventh day postoperatively using the independent t-test

Postoperative evaluation times	Intramuscular dexamethasone		Oral dexamethasone		p-value
	Mean	Std. Deviation	Mean	Std. Deviation	
1^st^ day	32.076	2.018	30.423	1.942	0.004
3^rd^ day	33.692	1.543	32.692	1.849	0.039
7^th^ day	38.538	2.831	37.653	3.440	0.316

The mean value of the VAS score for evaluation of pain for intramuscular dexamethasone on day one was found to be 6.884, and for oral dexamethasone, it was 7.961, and the difference between them was found to be statistically significant using the independent t-test. Similarly, for day two and day three, the mean value of the VAS score for intramuscular dexamethasone was 5.923 and 5.269, respectively, and for oral dexamethasone, it was 7.230 and 6.576, respectively. The statistical significance between the interventions on these two days was less. The postoperative pain scores using intramuscular dexamethasone and oral dexamethasone were similar and thus, can be used alternatively to each other based on the situation (Table 5).

**Table 5:** Comparison of pain using mean visual analog scale scores between intramuscular dexamethasone and oral dexamethasone on the first, second, and third day postoperatively using the independent t-test

Postoperative evaluation times	Intramuscular dexamethasone		Oral dexamethasone		p-value
	Mean	Std. Deviation	Mean	Std. Deviation	
1^st^ day	6.884	0.816	7.961	0.773	0.000
2^nd^ day	5.923	0.744	7.230	0.764	0.000
3^rd^ day	5.269	0.533	6.576	0.856	0.000

## Discussion

Therapeutic or prophylactic indications make odontotomy of the impacted third molar the commonest procedure performed by an oral & maxillofacial surgeon [13,14]. Like any surgical procedure, surgical removal of the impacted third molar is also associated with postoperative sequelae, such as pain, swelling, and trismus [15,16].

Due to the rich vascularization of the maxillofacial area, the inflammatory response tends to be more pronounced [17,18]. The severity of these postoperative sequelae is dependent on so many factors such as individual physiologic response to the procedure, amount of tissue trauma, duration of surgery, and manipulation, among many others [15,19].

To reduce tissue inflammatory mediators after surgery, corticosteroids are a well-known adjuvant. Dexamethasone is a long-acting, highly selective synthetic corticosteroid with strong anti-inflammatory properties [19]. It has a basic glucocorticoid effect and is roughly 25 times more potent than hydrocortisone, which is short-acting and six times more potent than prednisone and methylprednisolone, as they are intermediate-acting and equal in strength to betamethasone [20,21]. The corticosteroid used should have minimal impact on mineralocorticoids and good biological action. Dexamethasone satisfies these requirements since it is more effective than methylprednisolone and does not have any mineralocorticoid activity. Its half-life is approximately 36 to 72 hours. Dexamethasone acts by reducing the production of leukocytes and macrophages at the site of postoperative inflammation, which stops prostaglandin synthesis. It also causes negative regulation of genes for cytokines in macrophages, endothelial cells, and lymphocytes [22].

When a steroid injection is recommended in an outpatient setting, the intramuscular route is one of the most often utilized one. Studies on intramuscular injection of dexamethasone for third molar surgeries have revealed that this route can be effective if a single dose of dexamethasone is administered.

In our study, IM dexamethasone 8 mg was administered immediately following the removal of a lower third molar in the first group, and 8 mg of dexamethasone was given per oral in the second group. It demonstrated a considerable decrease in postoperative pain and edema.

We may correlate our findings with Elliott et al., who described the pharmacokinetic characteristics of 8 mg of intramuscular dexamethasone with an 8 mg of oral dose in 1996. In their investigation, drug bioavailability for the oral route was 72%, compared to 100% for the IM dosage. The terminal half-lives were similar across both groups, and the maximum plasma concentration for the oral dosage was 65% of that of the IM dose. Serum concentrations were maintained irrespective of the route of administration [21].

Corticosteroids must be administered in a dose greater than the physiologic levels released by the body to be effective. Cortisol's average daily output is 15 to 25 mg, but during times of crisis, it can reach 300 mg. A dose above this threshold would result in a higher inhibition of the inflammatory process than what the body already manages. After surgery, postoperative edema peaks 49 to 72 hours later. Therefore, if given in a single dose, glucocorticoids should have a longer duration of action [19]. Dexamethasone, being a long-acting glucocorticoid, has a half-life of around 36 to 72 hours and a potency that is 20 to 30 times more than that of natural corticosteroids while having a less immunosuppressive impact [21,22].

According to earlier research on dexamethasone and postoperative pain, individuals who took the medications per oral noticed a markedly reduced level of postoperative clinical pain and swelling. Schmelzeisen and Frolich demonstrated that the patient who got dexamethasone consumed less analgesics [4].

The present study aimed at comparing the effects of 8 mg dexamethasone IM (deltoid muscle) injection and 8 mg consumption of dexamethasone per oral immediately in the postoperative period on the day of the third molar surgery. This study used a case-control design with bias control and patients with bilateral lower third molars having similar difficulty index.

The parameters evaluated for this study were pain, swelling, and trismus. In our study, the amount of pain experienced by the patient was recorded using VAS. In terms of severity of pain, less of a statistically significant difference existed between both interventions. The mean value of the VAS pain score on day one for intramuscular dexamethasone was found to be 6.88, and for oral dexamethasone, it was 7.96. These results indicate that the oral dexamethasone group had a greater pain score than the intramuscular dexamethasone group on the first postoperative day, which may have been due to the maximum plasma concentration of oral dexamethasone (8 mg) being 65% than that of intramuscular dexamethasone (8 mg). Therefore, we recommend patients receiving dexamethasone orally to take oral analgesics for the initial two days. On postoperative day two and day three, both groups produced almost similar pain scores.

Overall, the results of the present study showed a reduction in the severity of the above-mentioned parameters at the test site on the first, third, and seventh days postoperatively. The result suggests that the measures of facial swelling for the intramuscular and oral dexamethasone groups were almost equivalent at all time points, and the outcomes were comparable. Trismus was evaluated by measuring the interincisal distance using a vernier caliper on the first, third, and seventh postoperative days. Both groups demonstrated the trismus' statistical relevance. The average values for each intervention group are quite comparable to one another, indicating that both of them may be employed to get effects that are expected to be similar. The parameters used in this study returned near to the normal baseline values on the seventh postoperative day in both oral and intramuscular dexamethasone groups.

Our results were in correlation with the previous study of Boonsiriseth et al. [4], who emphasized that no significant difference was found between the 8 mg dexamethasone given as an IM injection and the 8 mg oral dexamethasone. Both groups reported positive effects on all three parameters. They concluded that both forms of dexamethasone can be used post-third molar surgery [3].

As per our study, in terms of edema and trismus, there was less of a statistically significant difference between the two interventions. Comparatively, the mean VAS pain score on day one for intramuscular and oral administration of dexamethasone was 6.88 and 7.96, respectively. These findings show that on the first postoperative day, the oral dexamethasone group had a higher pain score than the intramuscular dexamethasone group, which may have been because the maximum plasma concentration of oral dexamethasone (8 mg) was 65% than that of intramuscular dexamethasone (8 mg).

The present study demonstrated that oral dexamethasone (8 mg) is a successful substitute for dexamethasone intramuscular injection (8 mg). Oral dexamethasone (8 mg) can be prescribed along with analgesics in the initial two postoperative days. On days two and three following the surgery, both groups had essentially equal pain scores. The use of the intramuscular injection may be a discomforting and painful experience for the patient. On the other hand, oral dexamethasone is relatively straightforward, less intrusive, painless, easy for the surgeon and for apprehensive patients, and provides a reasonably priced remedy for the agony usually connected with the extraction of impacted lower third molars.

## Conclusions

To sum up, oral dexamethasone is an effective alternative to intramuscular dexamethasone. It can be given after surgical extraction of the third molar, which will aid in the postoperative management of pain, swelling, and trismus. Oral dexamethasone is comparatively simple, less invasive, painless, and easy for the surgeon and for apprehensive patients, and it offers a cost-effective solution for the suffering often associated with the extraction of impacted lower third molars.
